# Microbial Desalination Cell for Sustainable Water Treatment: A Critical Review

**DOI:** 10.1002/gch2.202300138

**Published:** 2023-09-22

**Authors:** Soheil Aber, Zhining Shi, Ke Xing, Raufdeen Rameezdeen, Christopher W. K. Chow, Dharmappa Hagare, Tanu Jindal

**Affiliations:** ^1^ Sustainable Infrastructure and Resource Management (SIRM) UniSA STEM University of South Australia Mawson Lakes SA 5095 Australia; ^2^ School of Engineering, Design and Built Environment Western Sydney University Penrith NSW 2751 Australia; ^3^ Amity Institute of Environmental Toxicology, Safety and Management Amity University Noida 201303 India

**Keywords:** bioelectrochemical system, brackish water, saline water, scale up, sustainability

## Abstract

In view of increasing threats arising from the shortage of fresh water, there is an urgent need to propose sustainable technologies for the exploitation of unconventional water sources. As a derivative of microbial fuel cells (MFCs), microbial desalination cell (MDC) has the potential of desalinating saline/brackish water while simultaneously generating electricity, as well as treating wastewater. Therefore, it is worth investigating its practicability as a potential sustainable desalination technology. This review article first introduces the fundamentals and annual trends of MDCs. The desalination of diverse types of solutions using MDCs along with their life cycle impact assessment (LCIA)  and economic analysis is studied later. Finally, limitations and areas for improvement, prospects, and potential applications of this technology are discussed. Due to the great advantages of MDCs, improving their design, building materials, efficiency, and throughput will offer them as a significant alternative to the current desalination technologies.

## Graphical abstract

After an initial focus on the annual research trends of microbial desalination cells and their background theory and information, this article discusses the areas of improvement and potential applications of this technology. This article concludes that by amending microbial desalination cell construction and components, it will be a sustainable desalination technology with important agricultural and industrial applications.

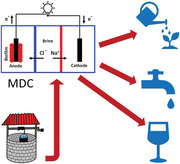

## 1 Introduction

With 2–15 kW h energy consumed^[^ ^1^ ^]^ and 6.7 kgCO_2_eq greenhouse gases produced^[^ ^2^ ^]^ for every cubic meter of fresh water produced, the conventional desalination units are highly energy intensive. High energy consumption, brine production, and CO_2_ emission are the main disadvantages of conventional desalination technologies. Due to the cost and operation complexity issues, these technologies cannot meet the needs of rural and outback areas where decentralized water treatment systems are better alternatives. Decentralized systems are smaller and more cost‐effective and have simpler technology compared with their centralized counterparts. For desalination purposes, microbial desalination cells (MDCs) have the potential to be utilized as decentralized systems and replace commercial reverse osmosis (RO) units.

MDC is a bioelectrochemical system (BES). BESs combine electrochemistry and biology and introduce new routes for chemical reactions in plenty of applications such as environmental remediation. Microbial fuel cell (MFC), MDC, microbial electrolysis cell (MEC), microbial reverse electrodialysis cell (MRC), and microbial Electrosynthesis (MES) are examples of BESs. While MES is basically designed for H_2_ production, it can produce methane as well. A review article by Pawar et al.^[^ ^3^ ^]^ investigated the operations, materials, and configurations used in MECs for electromethanogenesis, and the application of MEC for H_2_ production was studied by Son et al.,^[^ ^4^ ^]^ where they modified a cathode electrode by nickel powder, activated carbon, and polytetrafluoroethylene to make a Ni/Activated carbon/PTFE (polytetrafluoroethylene) cathode and obtained a hydrogen production rate of 1.88 L L^−1^ d^−1^, hydrogen purity of 97.5%, coulombic efficiency of 124%, and energy efficiency of 216%. The combination of reverse electrodialysis (RED) stack with MFC makes MRC.^[^ ^5^ ^]^ REDs use the concentration gradient between two salt solutions and ion movement through ion exchange membranes in order to generate electricity. When MFC is integrated with RED, it reduces the over‐potential on electrode surfaces, enhancing the generated power density. MES encompasses biocatalysts in the cathode chamber which consumes the electrons provided by a power source and converts CO_2_ to useful products like acetate and butyrate.^[^ ^6^ ^]^ By providing a new way of CO_2_ consumption, this technology can be of help in mitigating climate change and global warming.

**Figure 1:**
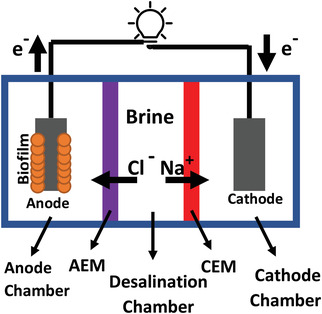
The schematic of a simple three‐chamber MDC.

MDCs, which emerged through the combination of electrodialysis cells and MFCs, have attracted a lot of attention in recent years due to their potential of being an environmentally friendly technology. MDC can simultaneously treat wastewater, desalinate saline water, and produce electrical energy at a low cost.^[^ ^7^, ^8^ ^]^ MDCs remove up to 99% of the salts of saline water and produce more energy than what they require.^[^ ^9^ ^]^ A simple MDC consists of an anode and a cathode chamber, an anion exchange membrane (AEM), and a cation exchange membrane (CEM). The anode and cathode are connected using an external circuit (**Figure** **1**). In this cell, the middle chamber produces fresh water as a result of the attraction of ions in the electric field toward electrodes. Furthermore, the organic molecules of the anolyte are anaerobically degraded in the anode chamber by the electrogenic bacteria, generating electrons which are transferred into the cathode chamber to reduce a terminal electron acceptor such as dissolved O_2_. Unlike the cathode chamber, the reactions in the anode chamber are sensitive to pH, and hence require the pH to be maintained ≈7.

*Geobacter sulfurreducens* and *Shewanella oneidensis* are two well‐known members of electrogenic bacteria.^[^ ^10^ ^]^ *Geobacter sulfurreducens* converts acetate to carbon dioxide and its growth is supported by electrode reduction.^[^ ^11^ ^]^ *Shewanella* species uses riboflavin as a mediator compound to enhance electron transfer from the bacteria to the electrode surface.^[^ ^12^ ^]^ Equation 1 demonstrates the glucose oxidation reaction in the anode chamber. Equations 2 and 3 show the four‐ and two‐electron oxygen reduction reaction (ORR) routes in the cathode chamber, respectively. The first route produces better coulombic efficiency, and therefore is the preferred mechanism. Four mechanisms are involved in the transfer of electrons from bacteria to the anode surface.^[^ ^13^ ^]^ The first uses artificial electron shuttles like neutral red. Shuttles are moving redox organic electron mediators that receive electrons inside the bacterial cell and transfer them to the anode surface. The second mechanism utilizes natural shuttles, like flavins and pyocyanin, secreted by electrogenic bacteria. Redox‐active proteins like cytochromes that are located on the outer surface of the cell membrane and transfer electrons to the electrode surface upon bacteria‐electrode close contact, present the third mechanism. Electron transfer from bacteria to the electrode surface through conductive pilis (nanowires) is the fourth mechanism.

(1) $$C_{6}H_{12}O_{6}\;+6H_{2}O\;\to \;6{\mathrm{CO}}_{2}\;+\;24H^{+}\;+\;24e^{-}$$

(2) $$O_{2}\;+\;2H_{2}O\;+\;4e^{-}\;\to \;4OH^{-}$$

(3) $$O_{2}\;+\;H_{2}O\;+\;2e^{-}\;\to {\;\mathrm{OH}}^{-}\;+\;{\mathrm{HO}}_{2}^{-}$$

Although there has been a plethora of review papers on MDCs over the recent decade,^[^ ^7^, ^9^, ^14^, ^15^, ^16^ ^]^ they all have fallen short in examining the development and utilization of MDC technology toward proposing real‐world applications. Hence, evaluating the potential utilization of MDC as an alternative technology to conventional desalination methods is essential. The present work will be casting light on the following key questions:

- What is the current status of MDC technology?
- What factors prevent the industrial utilization of MDCs?
- How can these barriers be removed?
- Where are the potential applications of the improved MDC technology?

This review first presents its method and data sources in Section 2, followed by examining the state of the extant literature on MDCs in relation to applications, life cycle impact assessment (LCIA), economic analysis, and scale‐up studies in Section 3. Finally, section 4 discusses the challenges encountered by MDCs, presents the potential solutions for easing the limitations, and offers potential areas for future research and some real‐world applications of MDCs.

## 2 Data Sources for Review

In order to access inclusive literature information, the Google Scholar database was used and the exact phrase “microbial desalination cell” was searched in document titles, in February 2023. To avoid the possible loss of relevant results no additional search term was used. All sources including journal articles, conference proceedings, academic books, theses and dissertations, abstracts, preprints, and technical reports were searched and only the patents and citations were excluded. No publication date limit was imposed.

The mentioned literature search resulted in a total of 205 papers from which review articles, non‐English, and inaccessible results were removed. To further narrow down the search results, the abstract and full text of the selected documents were analyzed to find those with similar trends and subjects, and therefore, only the representative ones were selected for the review. As a result, ≈56 documents were used for this review, and their full content including abstract, main text, images, and tables was analyzed in detail.

**Figure 2:**
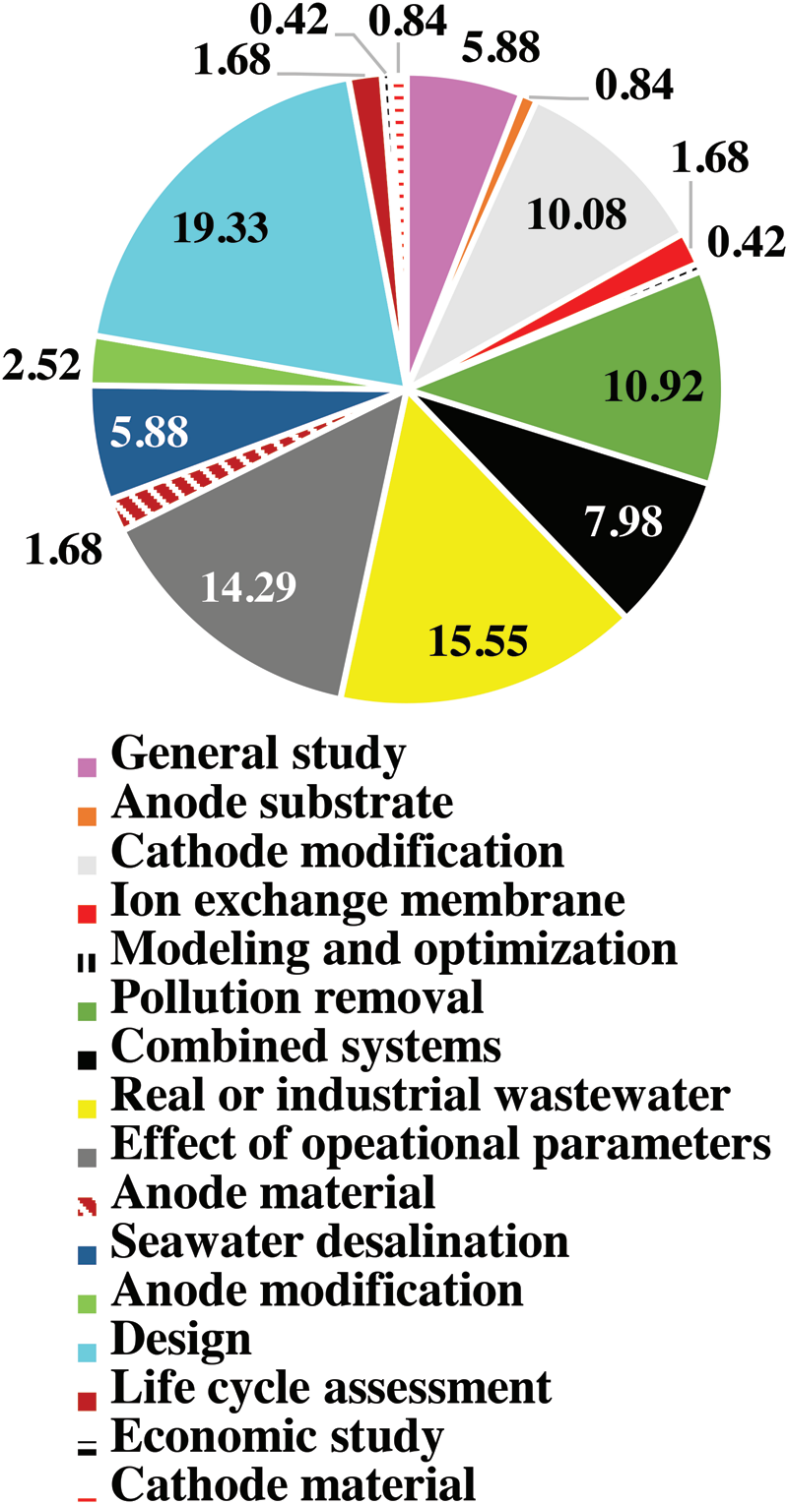
The distribution of main research subjects on MDCs.

**Figure** **2** plots the information collected as stated above after screening for patents, review articles, citations, non‐English articles, and those articles that were not accessible, without eliminating the documents with similar trends and subjects. The main ideas of the documents were inferred mostly from their titles while the content was also used when necessary. As can be seen in this figure, most of the published research (19.3%) has been focused on developing better designs and configurations of MDCs to improve their capabilities like desalination, power generation, and chemical oxygen demand (COD) removal. In addition, studies regarding real or industrial wastewater, and the effect of operational parameters attracted considerable attention with 15.6% and 14.3%, respectively. Removal of various pollutants in MDCs and modifying their cathodes have also been research focuses. Application of hybrid MDC systems, general studies, and seawater desalination have also been of interest (8%, 5.9%, and 5.9%, respectively). Anode modification, investigating the effect of the ion exchange membrane and anode material, life cycle assessment, exploring the effects of anode substrate and cathode material, modeling and optimization, and economic studies have been other subjects of interest for MDC researchers.

**Figure 3:**
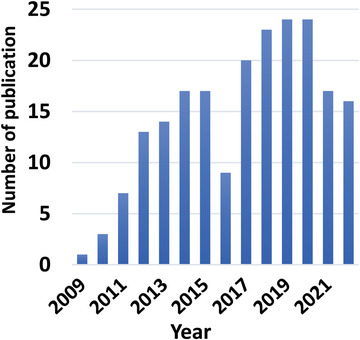
The annual distribution of MDC publications.

**Figure** **3** represents the annual number of MDC publications until 2022, which was created using the information collected through a search using the term “microbial desalination cell” in document titles in the Web of Science database. In this investigation, all kinds of publications were taken into account. As can be seen, MDC research started in 2009 with an upward trend for the majority of the years. In general, considering the low number of MDC publications compared with other topics and the urgent need for alternative desalination technologies, this topic can still be considered a fresh area.

**Figure 4:**
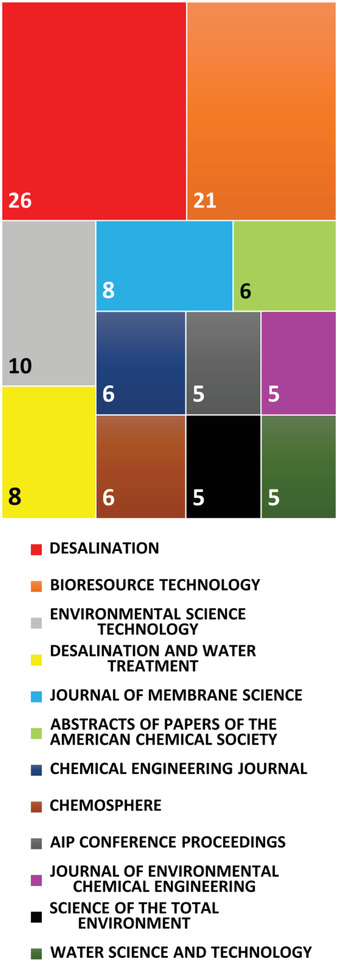
The top resources of MDC research based on the number of publications between 2009 and May 2023.

**Figure** **4** demonstrates the contribution of top scientific resources in disseminating the results of MDC research. As presented, “Desalination”, “Bioresource Technology, and ‘Environmental Science Technology” with 26, 21, and 10 documents, respectively, published the highest number of MDC works until May 2023. The information presented in this figure was extracted from the Web of Science and curated as described for Figure 3.

## 3 Current Research on MDC

### 3.1 MDC Applications

**Table** **1** shows the summary of a literature review on MDC applications. As can be seen here, MDCs have the flexibility to be used for various kinds of saline solutions such as brine produced by a desalination plant, diluted industrial wastewater, real geothermal water, and seawater.^[^ ^17^, ^18^, ^19^, ^20^ ^]^ They produce acceptable treatment results with a desalination performance of up to 97.4%^[^ ^21^ ^]^ and a COD removal efficiency of up to 97.8%.^[^ ^20^ ^]^ Moreover, they can remove toxic elements such as arsenic^[^ ^17^ ^]^ and boron^[^ ^22^ ^]^ from water. They have the capability of producing a power density of 566.1 mW m^−2[^ ^20^ ^]^ or an operational voltage of 710 mv^[^ ^23^ ^]^ together with desalination and wastewater treatment. Generally, MDC is an interesting technology, and with further research, it has the potential to emerge as a significant alternative method for water desalination.

**Table 1:** Summary of MCD applications for various types of water sources.

Study aims	Saline water type	Outcomes	Limitations
– Finding the effect of replacing external resistance with a power supply, – Finding the role of biofilm in the anode chamber.^[^ ^18^ ^]^	– The brine produced by a desalination plant in Italy (Sicily Region).	– 62% of the total salt was removed after 96 h, – MDC helps in energy saving since recycled metals decrease the need for mining, – Developing biofilm in MDC reduces energy consumption for desalination compared with MDCs with external potential supply or without biofilm.	– Using external potential supply increases the cost of the system operation.
– Investigation of ion transportation behavior.^[^ ^1^ ^]^	– NaCl solution, – Synthetic seawater, – Artificial seawater.	– Concentration and composition of the salt solution influence the ion transportation and fouling behavior, – Low efficiency of MDCs relates to low salt transfer and ion diffusion coefficient values.	– Multivalent ions tend to deposit on CEM, – Biofouling and inorganic deposition occur on AEM, – K^+^ significantly back diffuses from the catholyte to the desalination chamber.
– Simultaneous treatment and desalination of diluted industrial wastewater in a multi‐stage MDC.^[^ ^20^ ^]^	– Diluted industrial wastewater (COD: 8723 mg L^−1^, conductivity: 24 612 µS cm^−1^).	By applying an external voltage of 1 V, the following results were achieved: – Maximum power density of 566.1 mW m^−2^, – Removal efficiencies of 97.8%, 90.6%, and 98.4% for COD, total nitrogen (TN), and ammonium (NH^4+^–N), – 31.6% reduction in electrical conductivity.	– The addition of an external potential supply increases the cost of the system operation, – Flow from the cathode to the anode reduces MDC's efficiency by increasing oxygen concentration in the anode chamber.
– Simultaneous organics, sulfate, and salt removal.^[^ ^24^ ^]^	– Acetate and sulfate solutions.	– Sulfate and COD removal of 72% and 88%, – COD removal in sulfate‐fed MDC was higher than in acetate‐fed MDC.	—
– Investigation of MDC capability in Arsenic removal.^[^ ^17^ ^]^	– Synthetic arsenic solution (100–600 µg L^−1^) in deionized water, – Real water sample from the Bardsir region in Kerman, Iran, – Synthetic wastewater.	– The optimal conditions for Arsenic removal were: 600 mg L^−1^ As, 6 mg L^−1^ dissolved oxygen (DO), the temperature of 25–30 °C, and a retention time of 120 min, – Arsenic removal efficiency in the synthetic samples of As, real water, and synthetic wastewater were 75, 68, and 56%, respectively.	—
– Boron and COD removal.^[^ ^22^ ^]^	– Real geothermal water from geothermal power plant wells in Izmir, Turkey.	At optimum conditions, the following results were achieved: – A maximum boron removal efficiency of 55.5%, – COD removal efficiency of 91.5%, – Power density of 9 mW m^−3^.	– In the three‐dimentioanl (3D) anode, the cubic carbon particles are placed in a cube with a perforated surface. The probable blockage of the surface holes causes the system to stop working.
– Analysis of the electrochemical behavior of a three‐chamber MDC.^[^ ^19^ ^]^	– Brackish water (NaCl, 7 g L^−1^), – Seawater.	– Higher energy production lowers freshwater production and vice versa, – Salt removal efficiency of 93%, – Coulombic efficiency of up to 84%.	– In this study, ferricyanide was used as the catholyte. Compared with O_2_, it poses higher toxicity and cost.
– Investigating the efficiency of ozone as an electron acceptor in a three‐chamber MDC compared with O_2_ (O_3_‐MDC vs O_2_‐MDC).^[^ ^25^ ^]^	– NaCl solution (20 g L^−1^).	– 74% desalination for O_3_‐MDC vs 55.6% for O_2_‐MDC, – The power density of O_3_‐MDC was 11 times higher than O_2_‐MDC, – The maximum power density by O_3_‐MDC was 4.1 W m^−2^, – O_3_‐MDC had a lower internal resistance than O_2_‐MDC.	– The application of ozone as a cathodic oxidant may damage CEM, especially at high concentrations. It also poses higher operational costs, – Considering the high price and vulnerability of Pt to poisoning, this system is hard to use in practical applications.
– To study the efficiency of a three‐chamber MDC under different substrate strengths and external resistances.^[^ ^23^ ^]^	– Synthetic saline water (10 g L^−1^ NaCl solution).	– At substrate concentrations of 500, 1500, and 3000 mg L^−1^, COD removal efficiencies of 90.0, 92.3, and 53.4% were obtained, respectively, – With decreasing COD values, coulombic efficiencies (CEs) increased, – The best desalination efficiency was achieved at medium substrate strength.	—
– Ammonia recovery in anaerobic reactors with high ammonia levels using a submersible MDC.^[^ ^26^ ^]^	– Synthetic ammonia‐rich wastewater of an anaerobic reactor.	– Ammonia concentration decreased from 6 to 0.7 g‐N L^−1^ after 30 days, – An average recovery rate of 80 g‐N m^−2^ d^−1^, – Performance enhancement with an increase in initial ammonia concentration and a decrease in external resistance, – No negative effect of other cations on ammonia transportation was observed.	—
– Salt, sulfate, and nitrogen removal using a biocathode MDC.^[^ ^21^ ^]^	– High‐salinity mustard tuber wastewater.	– Salt (97.4%), sulfate (99.7%), and nitrogen (99.8%) can be removed, – The relative abundances of electrogenic bacteria in the anode and cathode were 16% and 15.1%, respectively, – The dominance of sulfate‐reducing bacteria in the anode, – A stable power output for 150 days.	– The use of Pt/C cathode is expensive and therefore, increasing the cathode surface for system scaleup is practically impossible, – Pt catalyst is vulnerable to poisoning and therefore, its application needs a catholyte with controlled composition
– Investigating the effect of temperature (12, 27, 45 °C) on MDC performance.^[^ ^27^ ^]^	– Artificial salt solution with a concentration of 20 g L^−1^ NaCl.	– Higher startup time is needed to reach stable performance at low temperature, – Average COD removal and desalination efficiencies of 46, 80, 78% and 9.4, 17.5, 22.7%, respectively, – Insignificant impact of temperature turbulence on COD removal and electricity generation but a negative effect on desalination performance.	– The temperature values selected in this study do not cover the entire applicable temperature range, – Different results can be obtained for the microbial community analysis based on the initial anodic microbial culture.

### 3.2 Life Cycle Impact Assessment and Economic Study of MDC Systems

LCIA investigates the environmental impacts that a product has during its full life cycle, starting from the production of those raw materials which are needed for its manufacturing, to the disposal of waste materials at the end of its life cycle.^[^ ^28^ ^]^ To help in the reduction of the environmental impacts of MDCs Zhang et al.^[^ ^29^ ^]^ analyzed their life cycle using a lab‐scale prototype system. While MDC has been known as an environmentally friendly technology, this study showed that its manufacturing and operation have significant environmental impacts. The electricity used for pumping water in the operation of MDCs has a contribution of 58.7% in global warming, while its manufacturing has a share of 22.7% as a result of the items involved such as the utilization of PTFE binder and ion‐exchange membranes. Therefore, maximizing the electricity produced by MDCs is vital for the improvement of their sustainability. They concluded that MFC and MDC technologies will be applicable for industrial applications after improvements.

The life cycle of MDCs was also assessed based on the emission of CO_2_, NO_X_, non‐methane volatile organic compounds (NMVOC), and SO_X_.^[^ ^30^ ^]^ It was found that CO_2_ emission by MDCs is higher than RO. Production of MDC components is responsible for the majority of emissions, hence, improving MDC design and using less carbon‐intensive materials might help in cutting or reducing CO_2_ emission. In terms of NO_x_, NMVOC, and SO_X_ emissions, MDC outperforms RO. To improve the net benefit of desalination by MDCs, freshwater production throughput needs to be increased.

Researchers compared MDC and RO technologies using life cycle and economic analyses as well.^[^ ^31^ ^]^ They aimed at investigating whether or not electricity production in MDCs makes them a more sustainable and economically favorable technology for long‐term desalination. Since the RO system used for comparison in this study was much larger than the studied MDC system, all results were normalized to one cubic meter of the produced water in Columbus, Ohio, USA. This study considered a timeline of 28 years of maintenance for MDC as it requires this long time to produce one cubic meter of fresh water. MDCs impact climate change, marine ecotoxicity, and fossil fuel depletion, therefore they are in need of improvements to be promoted to a more sustainable position. The conductive epoxy resin used to bond the copper wire to electrodes has the greatest impact on climate change (31%) with acrylic (29%) and chromium steel (28%) in the second and third places, respectively. Epoxy, at 32%, has the highest impact on fossil fuel depletion with acrylic as the second most important factor (28%). The copper wire had the main impact on marine ecotoxicity with 100% while other parameters had a total contribution of less than 0.03%. Chromium steel uses 98% of the total water consumed in the whole lifetime of MDC and therefore, is the most water‐intensive component. The prices of 16 components that were used for MDC manufacture, and the cost of assembly and maintenance processes were considered in cost‐benefit analysis. The net present value (NPV) for the production of 1m^3^ water by the MDC during 28 years was calculated to be ‐$28 281. The negative NPV clearly demonstrates that the MDC with the current design is not profitable for desalination. Epoxy resin was the most expensive item in MDC production, and the maintenance and upkeep of MDC imposed another considerable cost. During the assembly process, only less than one‐tenth of an epoxy resin tube was used and the remaining was wasted. By increasing the size of the MDC or using parallel MDC units, resin waste will be reduced, having a significant impact on dropping the total expense of the process. The investigated MDC operated in batch mode and thus, needed repetitions of feeding, cleaning, and re‐inoculating steps that increased maintenance and upkeep costs, meaning that designing continuous MDC systems will help in decreasing this type of process costs. Due to the long timeline of this study, the number of parts needing replacement increased for the production of one cubic meter of freshwater. Increasing the freshwater production throughput will decrease the process timeline and hence, the number of parts that should be replaced, which in turn will reduce the total process cost. The meaningful difference in the dimensions of the studied RO and MDC systems justifies a part of the superiority of the RO system, hence, at large‐scale MDC applications, the results of the calculations should be different. Although MDC is not currently a sustainable technology, further innovation in its design will improve its position from a sustainability perspective.

### 3.3 Scaling up of MDCs

The ultimate goal of conducting MDC research is preparing it as a technology for real‐world application on a large industrial scale. Therefore, accomplishing the scale‐up studies is absolutely necessary to investigate the problems that an MDC faces on a larger scale. Zhang and He^[^ ^32^ ^]^ studied the scaling up of a combined MDC and post‐aerobic system for more than 10 months with the aim of simultaneous wastewater treatment and seawater desalination for a total liquid volume of 105 L. To increase the volume of the system they connected 30 tubular MDC modules which were hydraulically connected through the anode and desalination chambers. Two feeding modes, including a single stream mode (one stream flowed through all the MDCs) and a four‐feeding‐point mode (four streams), were used. The feeding method had a critical influence on system performance; the multi‐point feeding method generated a much higher current than the single feeding mode. Furthermore, the current generation in different modules was dramatically ununiform. The lower current generated by the first module in both single‐point and multiple‐point feeding modes was due to the oxygen content of the fresh feed solution that was largely consumed in this module. The presence of oxygen in the anode chamber has an inhibitory effect on the current generation, hence, they employed an additional PVC tube to the second group of modules to consume DO content and diminish this problem. The distribution of organic material in the modules had a significant role in the current generation which increased with the four‐point feeding mode compared with the single‐point feeding. Large internal resistance, short circuit, and dry cathode were some other reasons for the low current production in some MDC modules. The multi‐point feeding mode resulted in a higher desalination rate, but lower COD removal efficiency compared with the single‐point feeding state. Furthermore, the effect of applying external voltage on desalination efficiency and energy consumption was studied, demonstrating that applying an external voltage improved the salt removal efficiency. Although applying an external voltage adds an energy‐consuming step to the process, it significantly decreases the total energy required by reducing the need for catholyte recirculation. Bearing in mind that the catholyte recirculation consumes 58–100% of the total energy, reducing the catholyte recirculation rate to an optimum value is the best way for increasing energy efficiency. Lowering catholyte recirculation intensity from 18.2 to 8.9 L min^−1^, decreased the energy consumption for both COD and salt removal by almost 40%; the difference increased to higher than 60% by dropping it to 3.1 L min^−1^. Reducing the catholyte recirculation rate had no major effect on current generation, COD removal rate, and total dissolved solids (TDS) removal rate.

In view of the above discussion, scale‐up studies provide critical data about the operation of MDC systems in larger application scales. These types of investigations inform us about mass and energy transfer difficulties, safety issues, sustainability and cost‐related problems that MDCs face in industrial applications. Therefore, running scale‐up studies of MDCs in parallel with the bench‐scale investigations is of the utmost importance.

## 4 Discussion

### 4.1 Limitations and Areas for Improvement

As previously mentioned, although MDCs produce electricity and treat wastewater while desalinating a saline solution, they cannot be considered sustainable solutions for desalination purposes at the current stage of their technology in comparison with other well‐established technologies such as RO. They possess low desalination efficiencies. Although the desalination percentages reported in the literature are sometimes significant, they usually need much time to be achieved. On the other hand, the CEMs and AEMs that are used in MDCs are costly which makes the application of MDCs uneconomical. Furthermore, membrane blockage via biofouling or inorganic matter deposition has the ability to disturb MDC behavior by blocking ion transportation. Salt back diffusion is also a major problem that prevents the acceptable performance of MDCs by rebounding some separated ions to the desalinated stream. The applied configurations are also the cause of some deficiencies. In some configurations where solution flows from the cathode to the anode, the presence of O_2_ from the cathode suppresses the activity of electrogenic anaerobic bacteria in the anode chamber imposing an inverse influence on MDC performance. In special designs, anode material is filled inside a perforated cube. The holes of the cube surface are the passways of the anolyte to be in contact with the anode material. These kinds of designs are exposed to the risk of hole blockage and process disruption. Moreover, the toxicity of the catholyte to the environment and also to the cathode catalyst should be considered. For example, the application of ferricyanide as a terminal electron acceptor poses toxicity problems and higher costs compared with oxygen. Ozone as an alternative to oxygen might damages the CEM, especially at high concentrations, and leads to higher operational costs. The instability and high price of the cathode catalyst are other problems. Some MDC studies have used Pt as an efficient cathodic catalyst; however, its high price prevents increasing the cathode surface area for system scaleup. The vulnerability of the Pt catalyst to chemical poisoning should also be considered which limits the application of catholyte ingredients. The cost and environmental impacts of manufacturing MDC components are major barriers to their broader application. Taking into account the substantial advantages of MDCs, further improvements in factors like design, building materials, efficiency, and throughput will enable them to become sustainable desalination alternatives.

### 4.2 Potential Areas of Improvement for MDCs

The knowledge of how operational parameters influence MDC performance has a crucial role in their management to achieve better outcomes. Each parameter has an enhancing or reducing impact on the response or even sometimes produces the best result in an optimum amount. Additionally, the interaction between different parameters should be taken into account. The coming subsections review the role of various parameters, which can be under operator control, on MDC performance.

#### 4.2.1 Modification of Cathode

O_2_, the most accessible terminal electron acceptor agent in the cathode chamber, is freely available everywhere. However, ORR on general electrode surfaces suffers from slow kinetics and consequently, there is a serious need to improve the conditions in the cathodic chamber in order to enhance ORR and obtain higher efficiency in MFCs and MDCs. Various approaches are applicable for this purpose. Application of photosynthetic^[^ ^33^ ^]^ and bacterial biocathodes,^[^ ^34^ ^]^ cathode surface modification using layered double hydroxide compounds (LDHs),^[^ ^35^ ^]^ CuO/ZnO nanoparticles,^[^ ^36^ ^]^ MnO_2_,^[^ ^37^ ^]^ ZIF‐67/activated carbon,^[^ ^38^ ^]^ and pyrolyzed iron ethylenediamine tetraacetic acid^[^ ^39^ ^]^ are the instances of the efforts made to improve cathodic efficiency. Considering the similarity between MFCs and MDCs, the same strategies can be applied to both of them.

In a group of biocathodes, microorganisms grow on the cathode surface to promote the rate of consumption of the electrons coming from the anode chamber. In another class of biocathodes, photosynthetic microorganisms produce O_2_ to capture electrons, similar to what occurs in algae‐assisted MDCs; here a higher concentration of oxygen is maintained in the cathode chamber which improves electron capture and current generation. In a photosynthetic cathode MDC using *Oscillatoria* sp. algae,^[^ ^33^ ^]^ a power density of 31.1 ± 1.0 mW m^−2^ (34.6 ± 1.5 mW m^−2^ for control MDC) and desalination efficiency of 61.8 ± 0.5% (57.3 ± 1.0% for control MDC) at an initial salt (NaCl) concentration of 10 g L^−1^ was achieved. The control MDC used phosphate buffer solution (PBS) as the catholyte with no algae. It took a long time for the photosynthetic MDC to achieve the peak and stable voltage production condition, which may be attributed to the time necessary for the adaptation period. The produced power densities and desalination efficiencies were functions of the salt concentration. This study offers that the modification of the cathode chamber cannot always produce better results since it depends on many parameters, therefore, it is mandatory to optimize the operational conditions.

Using stronger oxidation agents in the cathode chamber also improves MDC performance. Ozone has a higher oxidation potential compared with oxygen, and therefore better captures electrons in the cathode chamber. An MDC with ozone in the cathode chamber (O_3_‐MDC)^[^ ^25^ ^]^ outperformed the conventional MDC (O_2_‐MDC). Desalination of a NaCl solution with a concentration of 20 g L^−1^ generated an open‐circuit output voltage of 628 mV versus 1331 mV, a maximum power density of 0.4 W m^−2^ versus 4.1 W m^−2^, and a desalination efficiency of 55.6% versus 74% for O_2_‐MDC and O_3_‐MDC, respectively. Also, the duration of each cycle for O_3_‐MDC was longer than O_2_‐MDC (≈94 and 66 h, respectively) which indicates a more stable current profile in O_3_‐MDC. Considering the higher oxidation potential of ozone (2.1 V) compared with oxygen (1.2 V), the reported improvements can be easily justified but the higher cost of ozone production should be taken into account when selecting the oxidation agent.

Chemical methods are also attractive approaches to cathode modification. For this purpose, the cathode surface is usually modified using a catalyst to improve ORR and finally, power production and desalination efficiency. Platinum is a well‐known and efficient cathodic catalyst that has been exploited in many studies on MFCs. It has also been used in MDC studies.^[^ ^40^ ^]^ However, it is not useful for application on large scales because of its high cost and instability due to sulfide poisoning.^[^ ^41^ ^]^ Extensive research is necessary to find durable and affordable alternatives for catalysts based on platinum (and other precious metals). Silver‐tin dioxide (Ag‐SnO_2_) composite^[^ ^42^ ^]^ is a catalyst used to modify a carbon cathode in a five‐chambered MDC. The electrochemical characterizations revealed that the modified cathode has better ORR kinetics which leads to a significant increase in desalination efficiency (72.6 ± 3.0% vs 57.9 ± 8.6%) and power output (1.47 W m^−3^ vs 0.9 W m^−3^). This example shows the power of chemical methods in carrying out positive changes in the cathode surface and a substantial increase in MDC efficiency.

According to what was stated above, applying different techniques of cathodic ORR improvement is an important area that can be extensively investigated to enhance MDC performance and obtain better desalination efficiency.

#### 4.2.2 Modification of Anode

As stated before, the anode of an MDC is the source of electrons made available via biocatalytic reactions. The electrogenic bacteria like *Geobacter* spp., *Shewanella* spp., *Alpha‐, Beta‐, Gamma‐, Delta‐, and Epsilon‐proteobacteria* ^[^ ^43^ ^]^ are immobilized as a biofilm on the anode surface where they can consume the organic matter and generate electricity. Anode modification, through improving biofilm preparation and attachment, can assist in developing more efficient MDCs.

Preparing the anode as separated carbon particles, called 3D carbonaceous electrodes, produces a macro‐porous structure and a high surface area resulting in better biofilm growth and high capacity for electron storage. Comparing 3D and 2D (two‐dimensional) anodes^[^ ^22^ ^]^ for boron and COD removal in a batch MDC showed that the 3D anode with a 64.9% boron removal efficiency outperformed the 2D anode with an efficiency of 61.3% while COD improvement was not significant (90.7% vs 90.3%, respectively). In this work, the anode was made as a cubic chamber with holes on its faces, containing small 3D carbon cube particles. Another 3D anode, made by coating a composite of carbon nanotube (CNT) and chitosan on a commercial polyurethane sponge,^[^ ^44^ ^]^ showed great durability and electrochemical activity in a three‐chamber MDC. The prepared composite generated a higher power density (1776.6 mW m^–2^) and desalination rate (16.5 mg h^−1^), compared with commercial carbon felt electrode (1210.0 mW m^–2^ and 13.7 mg h^−1^, respectively) under the same conditions. The promising results were obtained because of the improved growth of bacteria and easier mass transfer in the 3D porous structure and better charge transfer due to the presence of CNT.

A photo‐microbial desalination cell (PMDC) was made by modifying the anode (circular graphite flake) with nano hematite (α‐Fe_2_O_3_).^[^ ^45^ ^]^ Hematite is a photocatalyst with a small bandgap of 2.2 eV, absorbing light in ultraviolet and visible regions. One side of the anode was modified by nano hematite to be irradiated using a 35‐W xenon lamp as the sunlight simulator, and the other side was left unmodified for biofilm growth. At an initial salt concentration of 20 g L^−1^, a maximum current density of 8.8 A m^−2^, twice that of the unmodified MDC, was obtained and a salt removal efficiency of higher than 96% was achieved which was significantly higher than the regular MDC. The introduced PMDC was capable of using solar irradiation to increase cell efficiency. Considering that solar energy is free, this does not increase the process cost. The examples discussed above show that there are attractive approaches for anode modification to substantially improve MDC performance.

#### 4.2.3 Setting Hydraulic Retention Time

Hydraulic retention time (HRT) is the average time interval that the reactants stay inside a reactor and is a function of reactor volume and flow rate. The effect of HRT on desalination efficiency was investigated in an up‐flow MDC comprised of two concentric cylinders.^[^ ^46^ ^]^ The internal cylinder was the anode chamber separated by an AEM from the desalination chamber (the external chamber). The outer surface of the desalination chamber was made of a CEM which the catholyte was flowing on its surface. This study indicated that with increasing HRT in the range of 1–4 days, the conductivity of the salt solution and artificial seawater, after passing through the desalination chamber, decreased and a rise in TDS reduction was found since the higher HRT provided enough opportunity for ions to cross the membranes. A similar trend was found for desalination efficiency in a three‐chamber MDC in galvanostatic conditions^[^ ^47^ ^]^ with a power source connected to the MDC making the cathode a negative pole and the anode a positive one. Here, with a decrease in HRT from 30.1 to 2.4, the desalination efficiency decreased from 77% to 12% while the nitrate removal rate increased from 17 up to 131 mg (NO_3_–N) L^−1^ d^−1^. An HRT of 4.9 h was able to remove nitrate concentration and salinity down to a concentration close to the World Health Organization (WHO) threshold limits. As a conclusion, in multi‐analyte systems, an optimum HRT should be applied to obtain the desired concentration of the analytes.

#### 4.2.4 Changing Inter‐Membrane Distance and Membrane Thickness

Inter‐membrane distance has an important role in MDC systems because it impacts the solution resistance against electrical current in the cell. Ping and He^[^ ^48^ ^]^ studied the effect of inter‐membrane distance on the desalination performance of a bench‐scale MDC. The results of this study demonstrated that a decrement in the inter‐membrane distance from 2.5 to 0.3 cm at a constant flow rate decreased the HRT value. Interestingly, they found that the salt removal efficiency was the highest at an inter‐membrane distance of 0.5 cm with a salt concentration of 10 g L^−1^ or at 2.5 cm with a salt concentration of 30 g L^−1^. Moreover, with decreasing the inter‐membrane distance the specific desalination rate increased, as it became 12 and 7 times at the initial salt concentrations of 10 and 30 g L^−1^, respectively, when changing the inter‐membrane distance from 2.5 to 0.3 cm. Even at a fixed HRT of 6 h, a higher desalination efficiency was obtained by decreasing the inter‐membrane distance.

Furthermore, membrane thickness has a significant role on MDC performance as well. With different membrane thicknesses, ion penetration across the membranes will differ, changing the internal resistance of MDC and desalination efficiency. Desalination efficiency and power density were improved by a decrease in membrane thickness.^[^ ^49^ ^]^ An increase of 26% was achieved in the desalination efficiency with a thinner membrane (lab‐made membrane) compared with the thicker one (commercial membrane) in a solution containing 2 g L^−1^ acetate and 20 g L^−1^ NaCl. Furthermore, the power density was increased to 295 ± 27 mW m^−2^, although CE was constant at ≈53 ± 5%. It is important to note that these findings should not be completely correlated to the thinness of the lab‐made membrane, since its ion exchange capacity was higher, which is an important operational parameter influencing the obtained results.

As expressed, inter‐membrane distance and membrane thickness are two important parameters with significant impact on the quality of desalination using MDCs. Therefore, casting membranes with optimum thickness and considering optimal distance between membranes should be considered for better results.

#### 4.2.5 Adjusting Catholyte and Anolyte Composition

While some catholytes significantly improve MDC performance, they are not suitable for commercial use because of high cost or other limitations. For instance, PBS has been known as an advantageous catholyte solution but suffers from limitations such as high cost and standards related to phosphate discharge in the environment.^[^ ^50^ ^]^ Ferricyanide solution,^[^ ^51^ ^]^ acidified water,^[^ ^52^ ^]^ and pH‐neutral NaCl solution^[^ ^53^ ^]^ are other instances of catholytes used in MDC investigations. In a research,^[^ ^50^ ^]^ the effect of different catholyte solutions including a 10 mm PBS, a 100 mm NaCl solution, a solution comprised of 50 mm PBS+100 mm NaCl, and a biocatholyte was studied on MDC performance. The biocatholyte was the effluent taken from the primary clarifier of a municipal wastewater treatment plant with no filtration. The initial conductivity of the used saline solution was decreased from 54 ± 0.9 mS cm^−1^ to 25.5 ± 1.6, 21.1 ± 1, 16.4 ± 1.9, and 11.8 ± 1.6 mS cm^−1^, respectively showing better performance of the biocatholyte solution. The MDC using the biocatholyte generated its highest power density with 32.6 W m^−3^, higher than that of the saline buffer catholyte with 29.4 W m^−3^. Biocatholyte had the least internal resistance, which improved current density. It showed a COD removal and desalination rate of 80% and 0.4 g (NaCl) L^−1^ h^−1^, respectively. The biocatholyte solution is low‐cost, easily applicable, and provides a natural buffering feature. All the investigated catholyte solutions were able to reduce MDC cost as an important barrier against MDC commercialization.

The anode chamber has a pivot role in the performance of MDC systems because electrons are produced here by the electrogenic microorganisms consuming the organic content of the anolyte solution. The organic matter content and its biodegradability level, the intermediate compounds produced during the biodegradation, the nutrients present in the anolyte, the conductivity of the anolyte, and the mass transfer condition in the solution all have crucial impacts on MDC performance. MDC performance can be adversely affected by the back diffusion of the anolyte into the desalinated stream, a phenomenon that is impacted by the Donnan effect and molecular transport. To investigate the back diffusion of organic compounds from the anode chamber, acetate, paracetamol, and ibuprofen were added to the anolyte solution as model compounds.^[^ ^54^ ^]^ It was found that the Donnan effect was dominant when no current was generated. When MDC was fed with a 5 g L^–1^ salt solution in an open circuit condition, acetate, phosphate, and sulfate ions each showed 1.9 ± 0.7%, 10.3 ± 1.3%, and 1.8 ± 1.2% back diffusion, respectively. At a high initial salt concentration of 35 g L^−1^ and a short HRT of 1.0 d, phosphate and sulfate showed the highest values of back diffusion with 7.1 ± 1.2% and 6.8 ± 3.1%, respectively, because of the high concentration gradient. Organic compound back diffusion was also intensified with an increase in salt concentration gradient. Moreover, the molecular weight of the organics had a significant role; higher molecular weight and greater hydrophobicity of the organic matter helped AEM in retaining them and reducing their back diffusion problem.

It is worth noting that the COD of the anolyte solution is an important parameter in desalination by MDCs. In a study^[^ ^21^ ^]^ with domestic sewage as the anolyte solution with COD concentrations of 400, 900, and 1400 mg L^−1^, high‐salinity mustard tuber wastewater (MTWW) as desalination stream, and ultrapure water as the catholyte, the COD value of 900 mg L^−1^ generated more electricity. However, the highest desalination efficiency was obtained at 5.3 mg h^−1^ at a COD value of 400 mg L^−1^. This study also suggests that the COD of the anolyte solution controls the growth of electroactive bacteria in the anode chamber.

As a result, where possible, the composition of catholyte and anolyte solutions should be determined while considering many factors such as cost, environmental discharge standards, toxicity, concentration, and the molecular weight of solution constituents.

#### 4.2.6 Effect of Internal and External Resistances

The external resistance (*R_ext_*) influences the transfer of electrons from the anode to the cathode through the external circuit. Internal resistance (*R_int_*), however, is a function of the MDC configuration, type and specification of electrodes, membranes, electrolytes, and biofilm. Reducing the number of chambers, using ion‐exchange resins, decreasing the distances between membranes and electrodes, and using electroconductive materials to fabricate electrodes help in diminishing *R_int_*. In BESs, *R_ext_* control is usually easier than *R_int_*. Rahman et al.^[^ ^2^ ^]^ investigated the optimum range of *R_ext_* in an MDC. For this purpose, they studied the effect of *R_ext_* at different values of *R_int_*. At a high amount of *R_int_* (200 Ω), the role of *R_ext_* was insignificant, while, at low *R_int_* values, it was important. At a low *R_int_* value (67 Ω), the optimal *R_ext_* was found to be in the range of 1–69 Ω and resulted in the highest desalination rate (10.41–8.59 mg h^−1^). This study approved the superiority of the effect of *R_int_* on desalination, before *R_ext_* optimization. Moreover, *Jafary* et al.^[^ ^52^ ^]^ reported the internal resistance as a key design factor in quadruple microbial desalination cells (QMDCs) that should be controlled during MDC operation. Ragab et al.^[^ ^23^ ^]^ studied the desalination efficiency at different substrate strengths and *R_ext_* values. They investigated the effect of *R_ext_* in the range of 10–10000 Ω on COD removal and CE and noticed its significant influence on these parameters. It was demonstrated that at *R_ext_* = 1000 Ω, the continuous feeding of MDCs with substrate concentrations in the range of low to medium at short batch cycles produced the best COD removal, CE, and voltage compared with operating at long batch cycles and high substrate concentration. At *R_ext_* values higher than 500 Ω, the CE decreased considerably. At high *R_ext_* values, the produced voltage was high but low current, power density, and desalination efficiency were obtained. At *R_ext_* >1000 Ω, approximately the same desalination efficiencies were produced at different substrate concentrations, since the high amount of *R_ext_* limits the number of transferred electrons, as the main driving force for desalination.

In conclusion, when operating MDC systems, proper measures should be considered to optimize and adjust *R_int_ and R_ext_* values paying attention to their important and complex roles in the performance of BESs.

#### 4.2.7 Role of MDC Configuration

MDC configurations with the potential to influence the pH balance, internal resistance, mass transfer, and retention time in the cell have a crucial role in its performance for desalination, COD removal efficiency, and power generation. There have been numerous novel MDC configurations developed based on interesting ideas. The design of more efficient configurations for MDCs has been the most attractive topic of MDC research (19.3%) (Figure 2). Different types of MDCs have been extensively reviewed before.^[^ ^7^, ^55^ ^]^ The various configurations reported in the literature can be categorized as stacked,^[^ ^56^, ^57^ ^]^ multi‐stage,^[^ ^20^ ^]^ tubular up‐flow,^[^ ^58^ ^]^ polygonal,^[^ ^52^ ^]^ submerged,^[^ ^59^ ^]^ and flow^[^ ^60^ ^]^ MDCs.

Stacked MDCs have one cathode and one anode chamber, but more than one desalination chamber.^[^ ^56^, ^57^ ^]^ The anode and cathode electrodes have positive and negative charges, respectively, providing an electric field as the driving force for the movement of ions in the solution. Ions cross the membranes according to their permeability, leaving some chambers and accumulating in other ones. As a result, fresh water and very salty water streams are generated in this system.

Furthermore, the direction of the streams between the chambers can be adjusted in such a way that the desalination path length increases. By managing the distribution of the ions between the chambers, the production of some chemicals is also possible. As an example, Al Hinani et al., 2022^[^ ^61^ ^]^ produced HCl and NaOH through an innovative MDC design. The design, construction, and operation of the stacked MDCs are simple and economical, but they need a larger space and land surface to be installed. As a result of the simple shape and accessibility of their parts, stack MDCs are the best choice to examine new ideas for the modification of MDC electrodes, membranes, and structural materials.

Multi‐stage MDC^[^ ^20^ ^]^ is another configuration to be used for desalination and wastewater treatment purposes. Here, there are more than one series of anode, desalination, and cathode compartments. In a rectangular container, the series are consecutively repeated. Various types of electrical connections and solution streams between cathodes and anodes are possible. Broadly speaking, multi‐stage MDCs have the same advantages and disadvantages as stacked MDCs, and the only difference is the increased number of cathode and anode chambers which means that the space used for desalination decreases. On the other hand, this configuration reduces the distance between the desalination chamber and the cathode/anode, enhancing the desalination efficiency.

Tubular up‐flow MDC is another category of MDCs that usually includes nested cylinders.^[^ ^58^ ^]^ The inner cylinder is the desalination chamber and the outer is where the cathode and anode electrodes are located. An air cathode electrode may replace the regular cathode. The two chambers are separated by a cylindrical ion exchange membrane, half of which (upper or lower) is AEM, and the other half is CEM. With such a configuration, the ions move to the outer cylinder and fresh water is produced in the inner section. This configuration can be used as a stacked design in which a stack of membranes (AEMs and CEMs) is used, providing many desalination and concentration chambers.^[^ ^62^ ^]^ In stack tubular up‐flow MDCs, the innermost cylinder is usually home to the anode chamber and the cathode is an air cathode in the outermost layer. A unique aspect of tubular up‐flow MDCs compared with regular rectangular ones is the fact that they require less space to operate. Their membranes can be made as ready‐to‐use modules that can be easily replaced; however, the manufacturing and maintenance of tubular systems are generally more difficult than rectangular ones.

Another interesting design is that of polygonal MDCs. They are made of several lateral compartments around a central part.^[^ ^52^ ^]^ The lateral compartments contain anodes and cathodes, and the central chamber is used for desalination. The AEMs are situated between the desalination and anodic chambers while CEMs separate the cathodic and desalination chambers. With such a design, anions and cations are transferred from the desalination chamber to the anodic and cathodic chambers, respectively, and freshwater is produced in the central chamber. Another type of polygonal MDC was suggested by Ebrahimi et al., 2018.^[^ ^63^ ^]^ They proposed a similar configuration, but the central chamber housed the anode, and the lateral compartments acted as cathodes. Between the central anode and each lateral cathode, a room was created using a pair of AEMs and CEMs as the desalination chamber. By reducing the distance between the anode and cathode chambers with the desalination chamber, the electrostatic attraction and repulsion forces would be stronger, and consequently, the quality of the produced fresh water would improve.

Submerged MDCs^[^ ^59^ ^]^ can be used as a portable answer for desalination. They are immersed in saline solution and have a simple configuration, similar to Figure 1, where the external walls of the anode and cathode chambers are made of ion exchange membranes. With such a design, the cations of the saline solution are transferred into the catholyte, and the anions into the anolyte, and finally, the salt concentration in the saline solution decreases. This design is helpful when directing the saline water into a specific desalination facility is impossible.

Another category of MDCs is called flow MDCs^[^ ^60^ ^]^ where the wastewater flows first into the anode chamber, then into the cathode chamber, and afterward, into the middle membrane stack. In each pass, the wastewater is partially purified. Zuo et al., 2016^[^ ^60^ ^]^ used this technology to convert 82.4% of the wastewater to a dilute one with desalination and COD removal efficiencies of 93.6% and 97.3%, respectively. With a conductivity of 68 ± 12 µS cm^−1^ and turbidity of 0.4 NTU, the produced desalinated water was usable as boiler supplementary water or industrial cooling water. Almost all the salt, phosphorus, and most of the nitrogen were recovered in the concentrated solution. In this system, instead of having separate anolyte, catholyte, and desalination solutions, only one wastewater stream passes through all the chambers. It is worth noting however, that membrane fouling in this style of MDC is a serious problem when real wastewater is treated. Zuo et al. tried to prevent the fouling problem by installing a membrane in the cathode chamber to control the quality of the solution flowing through the desalination membrane stack.

With plenty of MDC configurations that we have at our disposal and after applying improvements in this technology, it will be feasible to choose the best arrangement for a specific application and achieve the quality standards, all the while meeting economic and sustainability measures.

### 4.3 Future Prospects

MFC and MDC are novel technologies with the potential of being used for various purposes. MFC is the technology of the future. It simultaneously removes organic pollution and produces electricity, therefore, any type of wastewater with organic contents can be utilized for electricity production to launch processes with low energy demand such as MDCs for desalination purposes. MDC is an MFC‐based technology. It is nominally an environmentally friendly technology but for its practical applications, some improvements are mandatory.

The introduction of alternative cost‐effective and environmentally‐friendly materials for membranes, electrodes, wires, glues, and the main body of the device; modification of electrode surfaces with economic and non‐poisonous catalysts to enhance the electrochemical reactions; using continuous mode instead of batch one; improving system design for reducing the construction and maintenance costs, and reducing the energy for pumping water and wastewater in the system are instances of innovations that the researchers in this field should think about to make MDC a more viable desalination technology. The provision of the proposed prerequisites would promote MDC to be a practical tool for dealing with desalination and other environmental issues. Potential fields of the applications of promoted MDC technology are suggested in the next section.

The global acceptance of emerging technologies is a matter of time. Usually, when they are first introduced, competition with traditional technologies is challenging or even impossible. In spite of that, with the onset of the scarcity of natural resources that conventional technologies rely on, novel technologies become more popular and find real‐world applications. These days, human beings live in an era of serious scarcity of water resources, which stems from climate change and an increasing population. As a consequence, the priority of recycling wastewater increases. It is easily forecasted that the need and acceptance of water produced using treated wastewater will rise. The novel hi‐tech treatment methods will play a substantial role in this situation. For instance, in the Czech Republic, a beer has been produced from recycled and purified wastewater in the South Bohemian village of Čížová.^[^ ^64^ ^]^ This is an example highlighting some of the changes that people must eventually face in their habits. Of course, tough standards should be established and observed for the application of treated wastewater. Some potential industrial‐scale applications of MDCs are mentioned in the following section. In a nutshell, MDC will find acceptance as a sustainable and economic desalination technology soon, subject to substantial improvements in its construction and design.

#### 4.3.1 Potential New Applications of MDCs

Wineries consume a large amount of water for different stages of wine production and as a result, discharge a large volume of wastewater with high salinity. The production of each liter of wine releases 0.2‐4 L of wastewater^[^ ^65^ ^]^ which can be treated and reused for purposes like irrigation, washing, and fire prevention. Saline treated wastewater used for irrigation will leave salt deposits on the soil. This is a clear example of a situation where MDCs can be helpful by removing the high salinity of the treated wastewater. Shani and Ben‐Gal^[^ ^66^ ^]^ found that sodium and chlorine accumulate harshly in shoots of vine and the plant's mortality is related to the level and time of salinity. Salinity reduces the transpiration and growth of the vine, beginning immediately when salinity is experienced. At lower salinity levels, a delay in mortality onset is observed and the death rate rises with increasing the salinity exposure time. Liu et al.^[^ ^65^ ^]^ studied winery wastewater treatment using MFC technology reporting an energy recovery of 0.04 kW h kgCOD removed^–1^ and an output power density of 290–410 mW m^−3^. Interestingly, this shows that winery wastewater treatment releases electricity that can be used for desalination in an MDC to make the recycled wastewater suitable for irrigation.

Furthermore, hydroponic agriculture needs water of suitable quality. The high salinity of irrigation water can harm plants and reduce their productivity. Increasing the salinity of the irrigation water reduced rind thickness and fruit mass by 8.80% and 5.7%, respectively for hydroponic mini watermelons in a floating hydroponic system, per unit increase in water conductivity (dS m^−1^).^[^ ^67^ ^]^ In a hydroponic system for basil production, using a saline nutrient solution (40 and 80 mmol L^−1^ NaCl) reduced water consumption, growth, phytomass production, and the absolute growth rate of basil.^[^ ^68^ ^]^ In regions with a high salinity of water resources, MDCs can help in relieving the salinity problem to establish hydroponic agriculture systems. It is obvious that the presence of a source of organic wastewater for electricity production in MDC systems is inevitable, which is fortunately accessible everywhere. The same approach can be considered to desalinate the irrigation water for parks and green areas.

There are other instances of the harmful impacts of salinity. The discharge of severely saline wastewater produced during Na_2_CO_3_ production into the environment results in a serious rise in the conductivity of water resources and destructive effects on agricultural activities. The large volume of the produced saline effluent and the high cost of its treatment are the major causes of this problem. This issue was seen in north‐western Iran^[^ ^69^ ^]^ and some other areas around the world. Plenty of research papers back the capability of MDCs for desalination purposes (Table 1), as a result, MDC technology can be used as a pretreatment step to make the saline water quality/volume suitable for economic treatment using RO units.

Approximately 3% of the total water on the earth is freshwater.^[^ ^7^ ^]^ The increasing need for freshwater together with a decrease in desalination costs proliferated sea water desalination plants all over the world. RO and multiple‐effect distillation (MED) are two known desalination technologies. In excess of 300 million people rely on fresh water from desalination plants.^[^ ^70^ ^]^ According to the Utilization Report of Seawater in China,^[^ ^70^ ^]^ 115 seawater desalination projects with a capacity of 1 573 760 m^3^ d^−1^ were in operation in China by the end of 2019, and ≈15 906 desalination plants were active in 2019 in the world producing over 95.4 million m^3^ of water and nearly 141.5 million m^3^ of brine per day.^[^ ^71^ ^]^ This huge amount of brine has serious effects on water sources, the ecosystem, and agriculture when discharged into the environment. Therefore, suitable technologies must be adopted to decrease the environmental impacts of seawater desalination plants. Again, MDC technology can play a vital role in this field to relieve the problems regarding brine disposal. The application of an MDC unit results in a decrease in the volume/concentration of the brine stream, helping in its easier disposal.

Natural pollutants like arsenic and boron which are present in irrigation water, transfer to the crops and thereafter into the human body through the food chain. Considering the adverse effects of such pollutants, their concentration in irrigation water should be controlled. Published papers on the removal of boron,^[^ ^22^ ^]^ arsenic,^[^ ^17^ ^]^ copper,^[^ ^72^ ^]^ chromium and lead,^[^ ^73^ ^]^ and nickel^[^ ^74^ ^]^ back the potential capability of MDCs for removing pollutants from water. Desalination of underground water in rural areas is another potential usage of this technology, where because of the low population, establishing large desalination facilities is not viable. Giving consideration to the above discussion, important areas of utilization can be allocated to improve MDC technology, which will be a useful tool for exploiting salty/contaminated water resources.

## 5 Conclusion

Overall, while MDC research commenced in 2009, it is still a fresh area because MDC is an immature technology in the current situation, and is not yet prepared to resolve the desalination problem in the real world. It requires extensive research on its areas of improvement to be amended as a competitive technology against the well‐known conventional methods. Further improvements in aspects such as their design, building materials, efficiency, and throughput will change them into an effective alternative for desalination along with wastewater treatment. Considering the advantages this technology potentially has, future amendments will make it suitable for a wide range of applications in agriculture, industry, and other fields.

## Conflict of Interest

The authors declare no conflict of interest.

## References

[1] H. Alhimali , T. Jafary , A. Al‐Mamun , M. S. Baawain , G. R. Vakili‐Nezhaad , Biofuel Res. J. 2019, 24, 1090.

[2] S. Rahman , A. Al‐Mamun , T. Jafary , H. Alhimali , M. S. Baawain , Water Sci. Technol. 2021, 83, 2389. 34032617 10.2166/wst.2021.145

[3] A. A. Pawar , A. Karthic , S. Lee , S. Pandit , S. P. Jung , Environ. Eng. Res. 2022, 27, 200484.

[4] S. Son , B. Koo , H. Chai , H. V. H. Tran , S. Pandit , S. P. Jung , J. Water Process Eng. 2021, 40, 101844.

[5] H. Kang , E. Kim , S. P. Jung , Int. J. Hydrogen Energy 2017, 42, 27685.

[6] M. Quraishi , K. Wani , S. Pandit , P. K. Gupta , A. K. Rai , D. Lahiri , D. A. Jadhav , R. R. Ray , S. P. Jung , V. K. Thakur , R. Prasad , Fermentation 2021, 7, 291.

[7] L. K. S. Gujjala , D. Dutta , P. Sharma , D. Kundu , D.‐V. N. Vo , S. Kumar , Chemosphere 2022, 288, 132386. 34606888 10.1016/j.chemosphere.2021.132386

[8] A. Z. Imoro , M. Mensah , R. Buamah , Water‐Energy Nexus 2021, 4, 76.

[9] H. M. Saeed , G. A. Husseini , S. Yousef , J. Saif , S. Al‐Asheh , A. Abu Fara , S. Azzam , R. Khawaga , A. Aidan , Desalination 2015, 359, 1.

[10] S. Jung , Int. J. Electrochem. Sci. 2012, 7, 11091.

[11] D. R. Bond , D. R. Lovley , Appl. Environ. Microbiol. 2003, 69, 1548. 12620842 10.1128/AEM.69.3.1548-1555.2003 PMC150094

[12] E. Marsili , D. B. Baron , I. D. Shikhare , D. Coursolle , J. A. Gralnick , D. R. Bond , Proc. Natl. Acad. Sci. USA 2008, 105, 3968. 18316736 10.1073/pnas.0710525105 PMC2268775

[13] R. Kumar , L. Singh , Z. A. Wahid , M. F. Md. Din , Int. J. Energy Res. 2015, 39, 1048.

[14] A. Al‐Mamun , W. Ahmad , M. S. Baawain , M. Khadem , B. R. Dhar , J. Clean. Prod. 2018, 183, 458.

[15] S. Sevda , H. Yuan , Z. He , I. M. Abu‐Reesh , Desalination 2015, 371, 9.

[16] M. Patel , S. S. Patel , P. Kumar , D. P. Mondal , B. Singh , M. A. Khan , S. Singh , J. Environ. Manage. 2021, 297, 113374. 34325367 10.1016/j.jenvman.2021.113374

[17] M. Malakootian , H. Mahdizadeh , A. Nasiri , F. Mirzaienia , M. Hajhoseini , N. Amirmahani , Desalination 2018, 438, 19.

[18] R. A. Nastro , E. Leccisi , M. Toscanesi , G. Liu , M. Trifuoggi , S. Ulgiati , Energies 2021, 14, 4453.

[19] M. Ramírez‐Moreno , A. Esteve‐Núñez , J. M. Ortiz , Electrochim. Acta 2021, 388, 138570.

[20] K. Zuo , J. Chang , F. Liu , X. Zhang , P. Liang , X. Huang , Desalination 2017, 423, 104.

[21] Z. Liu , P. Xiang , Z. Duan , Z. Fu , L. Zhang , Z. Zhang , RSC Adv. 2019, 9, 25189. 35528677 10.1039/c9ra04184b PMC9069894

[22] A. Y. Goren , H. E. Okten , Desalination 2021, 518, 115267.

[23] M. Ragab , A. Elawwad , H. Abdel‐Halim , Renewable Energy 2019, 143, 939.

[24] T. Jafary , W. R. W. Daud , S. A. Aljlil , A. F. Ismail , A. Al‐Mamun , M. S. Baawain , M. Ghasemi , Desalination 2018, 445, 204.

[25] A. Gholizadeh , A. A. Ebrahimi , M. H. Salmani , M. H. Ehrampoush , Chemosphere 2017, 188, 470. 28898779 10.1016/j.chemosphere.2017.09.009

[26] Y. Zhang , I. Angelidaki , Bioresour. Technol. 2015, 177, 233. 25496943 10.1016/j.biortech.2014.11.079

[27] M. Ragab , A. Elawwad , H. Abdel‐Halim , Desalination 2019, 462, 56.

[28] G. Finnveden , M. Z. Hauschild , T. Ekvall , J. Guinée , R. Heijungs , S. Hellweg , A. Koehler , D. Pennington , S. Suh , J. Environ. Manage. 2009, 91, 1. 19716647 10.1016/j.jenvman.2009.06.018

[29] J. Zhang , H. Yuan , Y. Deng , Y. Zha , I. M. Abu‐Reesh , Z. He , C. Yuan , J. Clean. Prod. 2018, 200, 900.

[30] N. R. Faze , G. M. Girme , T. A. Bower , A. D. Christy , B. R. Bakshi , American Society of Agricultural And Biological Engineers, Montreal, Quebec, Canada 2014, p. 141913148.

[31] N. R. Faze , Ph.D. Thesis , The Ohio State University, 2015.

[32] F. Zhang , Z. He , Desalination 2015, 360, 28.

[33] D. Bejjanki , K. Muthukumar , T. K. Radhakrishnan , A. Alagarsamy , A. Pugazhendhi , S. Naina Mohamed , Sci. Total Environ. 2021, 754, 142215. 32920416 10.1016/j.scitotenv.2020.142215

[34] X. Cao , P. Liang , X. Song , Y. Wang , Y. Qiu , X. Huang , Sci. China Technol. Sci. 2019, 62, 1703.

[35] R. Tajdid Khajeh , S. Aber , M. Zarei , Renewable Energy 2020, 154, 1263.

[36] R. Tajdid Khajeh , S. Aber , K. Nofouzi , Mater. Chem. Phys. 2020, 240, 122208.

[37] P. Zhang , K. Li , X. Liu , J. Power Sources 2014, 264, 248. 10.1016/j.jpowsour.2013.09.094 PMC3855397 24327797

[38] B. Koo , S. P. Jung , Chem. Eng. J. 2021, 424, 130388.

[39] X. Xia , F. Zhang , X. Zhang , P. Liang , X. Huang , B. E. Logan , ACS Appl. Mater. Interfaces 2013, 5, 7862. 23902951 10.1021/am4018225

[40] X. Chen , X. Xia , P. Liang , X. Cao , H. Sun , X. Huang , Environ. Sci. Technol. 2011, 45, 2465. 21322552 10.1021/es103406m

[41] R. Burkitt , T. R. Whiffen , E. H. Yu , Appl. Catal., B 2016, 181, 279.

[42] G. Anusha , M.d. T. Noori , M. M. Ghangrekar , Mater. Sci. Energy Technol. 2018, 1, 188.

[43] H. Zhou , X. Mei , B. Liu , G. Xie , D. Xing , Biotechnol. Biofuels 2019, 12, 133. 31164924 10.1186/s13068-019-1477-9 PMC6543681

[44] C.‐Y. Ma , C.‐H. Hou , Sci. Total Environ. 2019, 675, 41. 31026642 10.1016/j.scitotenv.2019.04.174

[45] Y. Liang , H. Feng , D. Shen , N. Li , Y. Long , Y. Zhou , Y. Gu , X. Ying , Q. Dai , Electrochim. Acta 2016, 202, 197.

[46] K. S. Jacobson , D. M. Drew , Z. He , Environ. Sci. Technol. 2011, 45, 4652. 21526816 10.1021/es200127p

[47] G. Puggioni , S. Milia , V. Unali , R. Ardu , E. Tamburini , M. D. Balaguer , N. Pous , A. Carucci , S. Puig , Sci. Total Environ. 2022, 845, 157236. 35810909 10.1016/j.scitotenv.2022.157236

[48] Q. Ping , Z. He , Desalin. Water Treat. 2014, 52, 1324.

[49] M. Mehanna , T. Saito , J. Yan , M. Hickner , X. Cao , X. Huang , B. E. Logan , Energy Environ. Sci. 2010, 3, 1114.

[50] A. Ebrahimi , G. D. Najafpour , D. Y. Kebria , Desalination 2018, 432, 1.

[51] D. A. Koomson , J. Huang , G. Li , N. Miwornunyuie , W. K. Darkwah , Renewable Energy 2022, 189, 1375.

[52] T. Jafary , A. Al‐Mamun , H. Alhimali , M. S. Baawain , M. S. Rahman , S. Rahman , B. R. Dhar , M. Aghbashlo , M. Tabatabaei , Renewable Sustainable Energy Rev. 2020, 127, 109855.

[53] Y. Zhang , I. Angelidaki , Biotechnol. Bioeng. 2015, 112, 1478. 25620722 10.1002/bit.25549

[54] Q. Ping , O. Porat , C. G. Dosoretz , Z. He , Water Res. 2016, 88, 266. 26512804 10.1016/j.watres.2015.10.018

[55] M. Zahid , N. Savla , S. Pandit , V. K. Thakur , S. P. Jung , P. K. Gupta , R. Prasad , E. Marsili , Desalination 2022, 521, 115381.

[56] X. Chen , H. Sun , P. Liang , X. Zhang , X. Huang , J. Power Sources 2016, 324, 79.

[57] A. Ziaedini , H. Rashedi , E. Alaie , M. Zeinali , J. Environ. Chem. Eng. 2018, 6, 5079.

[58] T. Jafary , A. Al‐Mamun , H. Alhimali , M. S. Baawain , S. Rahman , W. A. Tarpeh , B. R. Dhar , B. H. Kim , Desalination 2020, 481, 114358.

[59] Y. Zhang , I. Angelidaki , Water Res. 2013, 47, 1827. 23375601 10.1016/j.watres.2013.01.005

[60] K. Zuo , Z. Wang , X. Chen , X. Zhang , J. Zuo , P. Liang , X. Huang , Environ. Sci. Technol. 2016, 50, 7254. 27269411 10.1021/acs.est.6b00520

[61] A. Al Hinai , T. Jafary , H. Alhimali , S. Rahman , A. Al‐Mamun , Desalination 2022, 525, 115488.

[62] Y. Wang , A. Xu , T. Cui , J. Zhang , H. Yu , W. Han , J. Shen , J. Li , X. Sun , L. Wang , Chemosphere 2020, 248, 126028. 32018109 10.1016/j.chemosphere.2020.126028

[63] A. Ebrahimi , D. Yousefi Kebria , G. D. Najafpour , Chem. Eng. J. 2018, 354, 1092.

[64] Czech brewery rolls out first wastewater beer, https://english.radio.cz/czech‐brewery‐rolls‐out‐first‐wastewater‐beer‐8128614 (accessed: September 2022).

[65] T. Liu , A. V. Nadaraja , J. Shi , D. J. Roberts , J. Environ. Eng. 2021, 147, 04021043.

[66] U. Shani , A. Ben‐Gal , Am. J. Enol. Vitic. 2005, 56, 148.

[67] L. M. Gomes do Ó , A. M. W. Cova , A. D. de Azevedo Neto , M. G. Souza , A. L. Santos , H. R. Gheyi , Pesqui. Agropecuária Trop. 2021, 51, 67054.

[68] J. F. dos Santos , M. A. Coelho Filho , J. L. Cruz , T. M. Soares , A. M. L. Cruz , Rev. Ceres 2019, 66, 45.

[69] A. Asghari Moghaddam , N. Mahmoudi , J. Environ. Stud. 2008, 34, 15.

[70] X. Xiang , X. Liu , Desalination 2022, 533, 115734.

[71] C. Campero , N. J. Bennett , N. Arriagada , Geogr. J. 2022, 1, 231.

[72] Z. An , H. Zhang , Q. Wen , Z. Chen , M. Du , Desalination 2014, 346, 115.

[73] A. Gholizadeh , M. H. Salmani , A. A. Ebrahimi , S. S. Hosseini , M. H. Ehrampoush , M. Miri , A. Nikoonahad , H. Pasalari , Environ. Chem. Lett. 2018, 16, 1477.

[74] F. Mirzaienia , A. Asadipour , A. J. Jafari , M. Malakootian , Appl. Water Sci. 2017, 7, 3617.

